# Subjective-Physiological Coherence during Food Consumption in Older Adults

**DOI:** 10.3390/nu14224736

**Published:** 2022-11-09

**Authors:** Akie Saito, Wataru Sato, Akira Ikegami, Sayaka Ishihara, Makoto Nakauma, Takahiro Funami, Tohru Fushiki, Sakiko Yoshikawa

**Affiliations:** 1 Psychological Process Research Team, Guardian Robot Project, RIKEN, Kyoto 619-0288, Japan; 2 Field Science Education and Research Center, Kyoto University, Kyoto 606-8502, Japan; 3 San-Ei Gen F. F. I., Inc., Toyonaka 561-8588, Japan; 4 Faculty of Agriculture, Ryukoku University, Ohtsu 520-2194, Japan; 5 Faculty of the Arts, Kyoto University of the Arts, Kyoto 606-8501, Japan

**Keywords:** emotion, facial electromyography (EMG), food, subjective ratings

## Abstract

Background: Subjective-physiological emotional coherence is thought to be associated with enhanced well-being, and a relationship between subjective-physiological emotional coherence and superior nutritional status has been suggested in older populations. However, no study has examined subjective-physiological emotional coherence among older adults while tasting food. Accordingly, the present study compared subjective-physiological emotional coherence during food consumption among older and younger adults. Methods: Participants consumed bite-sized gel-type foods with different flavors and provided their subjective ratings of the foods while their physiological responses (facial electromyography (EMG) of the corrugator supercilia, masseter, and suprahyoid, and other autonomic nervous system signals) were simultaneously measured. Results: Our primary findings were that (1) the ratings of liking, wanting, and valence were negatively correlated with corrugator EMG activity in older and young adult participants; (2) the positive association between masseter EMG activity and ratings of wanting/valence was weaker in the older than in the young adult group; and (3) arousal ratings were negatively correlated with corrugator EMG activity in the older group only. Conclusions: These results demonstrate commonalities and differences in subjective-physiological emotional coherence during food intake between older and young adults.

## 1. Introduction

Coherence between emotional subjective experience and physiological responses is thought to contribute to psychological well-being [1,2,3,4], which is crucial to the quality of health and life, particularly in older adults. In addition, food evaluation is essential for older adults, who are more vulnerable to nutritional deficiencies [5,6]. It has been demonstrated that coherence between subjective responses to smells and physiological activity, assessed by corrugator electromyography (EMG) (bodily responses), is relevant to superior nutritional status in older adults, suggesting that subjective-physiological emotional coherence plays a critical role in consuming appropriate foods by older adults, which may enhance the quality of health and life in late adulthood [7].

Some recent studies have demonstrated that subjective-physiological emotional coherence was observed in young adults tasting foods [8,9]. In these studies, the participants’ subjective ratings and physiological signals of flavored gel-type foods were assessed while consuming these foods. The results demonstrated that the subjective ratings scores were negatively correlated with facial EMG activity of the corrugator supercilia and positively correlated with masseter and suprahyoid EMG, respectively [8,9]. Thus, these results indicate that when young adults perceive foods as unpalatable, they are more likely to demonstrate brow activity (frowning), and when they perceive foods as palatable, they are more likely to chew the food.

With regard to older adults, a relevant previous study examining the relationship between subjective ratings of odors and physiological activities (corrugator EMG) in older and young adults has reported no age difference in subjective-physiological emotional coherence [7]. Likewise, another study utilizing film clips as emotion-eliciting stimuli has demonstrated no difference in subjective ratings and corrugator EMG activity between young and older adults [10]. Although these previous studies of older adults imply maintenance of subjective-physiological emotional coherence in older adults during the food consumption, no studies have examined subjective-physiological emotional coherence in older adults during food consumption. It is therefore worth examining the magnitude of subjective-physiological emotional coherence among older adults during food consumption.

Additionally, although previous studies of older adults have demonstrated alterations in taste acuity with increasing age [6,11,12,13], such alterations do not appear to affect subjective reports of foods including hedonic liking of the foods in older adults [14,15]. It is, therefore, possible to estimate subjective responses to food in older adults and thereby examine subjective-physiological emotional coherence during food intake. Furthermore, increasing age is assumed to be associated with decreases in chewing efficiency [15,16,17], which might impact the strength of masticating food while older adults are consuming food. This age-related physical change should be considered when interpreting the results of investigating subjective-physiological emotional coherence during food intake among older adults.

In this study, we examined subjective-physiological emotional coherence during food consumption in older adults. To examine this issue, we assessed subjective ratings provided by older adults, while the older adults were eating bite-sized gel-type foods of different flavors, as used in studies of young adults [8,9]. These foods were highly likely to evoke different emotions, and the physiological activities of older adults were measured during consumption of the foods. Then, we compared the magnitude of subjective-physiological emotional coherence during food intake among older adults to that of young adults, who had undergone the same experimental procedures as those in the current study [9]. Considered the evidence of relevant previous studies that examined subjective-physiological emotional coherence in terms of subjective ratings and corrugator EMG activity [7,10], we hypothesized that subjective-physiological emotional coherence between subjective ratings and corrugator EMG activity in older adults would be comparable to that of young adults. Specifically, based on the findings from previous studies of young adults [8,9], we hypothesized that self-reports of hedonic experience (ratings of liking, wanting, and valence) of foods would be negatively correlated with corrugator EMG in older adults, similar to young adults. In contrast, given the fact that older adults have a lower chewing efficiency [15,16,17], we predicted weaker positive correlations between the subjective ratings and masseter and/or suprahyoid EMG among older adults than those of young counterparts.

## 2. Materials and Methods

### 2.1. Participants

Twenty-seven older Japanese adults (15 females and 12 males; mean age ± Standard Deviation (SD) = 69.7 ± 3.5 years; range: 65–78 years) were recruited from the community sample via advertisements. The participants were classified as older adults according to guidelines for bias-free language provided in the Publication Manual of the American Psychological Association (APA) (7 th Ed.). The participants were required to be >65 years old, be in the normal weight range (body mass index (BMI) < 30 kg/m^2^; mean BMI ± SD = 23.0 ± 3.3 kg/m^2^), and to have real teeth remaining. Only normal-weight participants were tested, as obese participants demonstrate distinctive food preferences. Although one additional female participant was tested, her data were not recorded and analyzed due to an equipment error. The sample size was determined based on an a priori power analysis using G*Power software (ver. 3.1.9.2; Dusseldorf, Germany) [18]. The subjective-physiological emotional coherence was analyzed using an independent *t*-test (older vs. young groups; two-tailed) with an effect size *d* of 0.8 (large), α level of 0.05, and power (1 − β) of 0.8. The results demonstrated that 26 participants were required in each group. All participants had fasted for more than 3 h before the experiments. The Japanese version of the Mini-Mental State Examination (MMSE) [19,20] demonstrated that the cognitive ability of all of the older participants was within the normal range (i.e., >24; mean ± SD = 29.0 ± 1.7). The data of a community sample of 29 younger (<40 years; mean age ± SD = 24.3 ± 5.2 years, aged 20 to 38) Japanese adult participants (16 females and 13 males) from a previous study [9], who completed an identical experiment, were analyzed to examine age differences. The young adult participants were non-obese (mean BMI ± SD = 21.9 ± 2.7 kg/m^2^), fasted (>3 h). Ethical approval for this study was obtained from the Ethics Committee of the Unit for Advanced Studies of the Human Mind, Kyoto University.

### 2.2. Stimuli

Nine flavors of bite-sized gel-type solid food materials (water-based gellan gum jellies) were prepared and presented to each participant, twice in total. We used gel-type foods as stimuli because their flavors and physical properties can be controlled and evaluated easily. In addition, gels can easily be consumed by older people. For simplicity, we used a single representative flavor component in each sample. The other three food items were prepared for practice. A total of 10% sucrose, 0.2% anhydrous citric acid, 0.03% trisodium citrate, 0.1% calcium lactate, and 0.35% or low-acetylated gellan gum (Kelcogel; San-Ei Gen F.F.I., Osaka, Japan) were contained in all stimuli. Additionally, nine specific flavoring agents, three of which are generally evaluated as negative, neutral, and positive hedonic qualities were contained in the stimuli. The agents were: 0.0010% isovaleric acid, 0.0005% (E)-2-nonenal, and 0.0010% indole for negative; 0.0100% phenethyl alcohol, 0.0100% acetoin, and 0.0100% 2,5-dimethylpyrazine for neutral; and 0.0200% vanillin, 0.0200% maltol, and 0.0200% ethyl butyrate for positive traits. All of the flavors were provided by San-Ei Gen F.F.I. These flavor compounds are utilized in common food products and are consumed as part of a normal diet. The concentrations of the flavoring agents were determined in preliminary experiments. Because food preferences generally differ among individuals [21], we wanted to examine individual-specific subjective-physiological coherence. By conducting preliminary experiments, we were able to determine the number of food stimuli, such that participants with a lighter appetite could be able to consume the prepared food materials.

Sucrose and low-acetylated gellan gum were mixed and poured into deionized water at the temperature of 90 °C, in a 500-mL glass beaker. And then they were stirred at 1300 rpm for 10 min at the same temperature to prepare the gel foods. Next, trisodium citrate, calcium lactate, and anhydrous citric acid were added to the solution. The solution was poured into plastic cups 65 mm in diameter and 25 mm in height, in which cylindrical glass molds (20 mm in diameter, 10 mm in height) are placed. The cups were sealed, heated at 85 °C for 30 min, and refrigerated for 1 h at 8 °C. The diameter and height of the prepared gels were 20 mm and 10 mm, respectively. We determined the fracture strain and force of these gels, using a 75-mm diameter aluminum plate, which compressed the gels on a metal stage, operating at a crosshead speed of 10 mm/s at 20 °C, and by measuring via a TA XT-2i texture analyzer (Stable Micro Systems, Surrey, UK). Consequently, we used the gel stimuli with a fracture force of 16.6 ± 0.6 N, and a fracture strain of 44.4 ± 2.3%.

### 2.3. Procedure

In the experiment, the older adult participants were required to provide subjective ratings of the foods in terms of liking, wanting, valence, and arousal, while we measured the facial physiological activity via EMG of the corrugator supercilia, zygomatic major, masseter, and suprahyoid muscles. The experiment was conducted in an electrically shielded soundproof chamber (Science Cabin; Takahashi Kensetsu, Tokyo, Japan), in which the room temperature was maintained at 23.5–24.5 °C, and monitored by a TR-76Ui (T&D, Matsumoto, Japan). Participants were tested individually. The experiments were controlled by Presentation 14.9 software (Neurobehavioral Systems, Berkeley, CA, USA) on a Windows computer (HP Z200 SFF, Hewlett-Packard Japan, Tokyo, Japan). Visual stimuli were presented on a 19-inch computer screen (HM903D-A; Iiyama, Tokyo, Japan).

Before starting the experiment, the participants were informed that subjective hedonic ratings and physiological responses would be measured while they ate food stimuli. In the experiment, the dish containing the nine food stimuli was placed on a table in front of a monitor. The dish was replaced with another dish containing nine different food stimuli during recess. All food stimuli had been prepared before the experiment for about 10 min. In order for participants to be able to eat the food using one hand in a predefined order, the food stimuli were placed in a row on 8-cm plastic disposable spoons. The main experiment (18 trials) was carried out with a short recess after half of the trials after three practice trials. The stimuli were presented pseudo-randomly, without repetition of the foods with the hedonic quality. The inter-trial interval varied between 20 and 30 s.

A small white cross appeared against a black background on the screen for 3 s, as a warning cue in each trial. During this period, the participants had to prepare to consume the food stimulus by holding the spoon close to their mouths. Then, a large red cross appeared for 10 s to signal the consumption period. As soon as the red cross appeared, the participants had to consume each food stimulus and keep chewing without swallowing during the presentation of the red cross. Subsequently, a rating display with four scales (liking, wanting, valence, and arousal) continued to be presented until the ratings were finished. During this period, the participants had to swallow the food and report their hedonic experiences during the consumption of each food stimulus by pressing the appropriate key. In the response panel, 9-point rating scales for each type of subjective rating were presented simultaneously in the fixed order of liking, wanting, valence, and arousal. The liking and wanting ratings were completed using these terms as labels and lines with numbers and wording indicating 1 (dislike) to 9 (like) and 1 (do not want to eat) to 9 (want to eat), respectively. These terms were presented as labels and numbers with images of self-assessment manikins [22] for valence and arousal ratings. The participants rinsed their mouths with mineral water after the ratings. We also performed exploratory measurements of other physiological responses to food, including the skin conductance response (SCR) and heart rate (HR), to evaluate correlations between subjective ratings and physiological measurements. Previous studies suggested that these physiological measurements are less likely to demonstrate robust correlations with subjective ratings in young adults [9,23].

### 2.4. Physiological Data Recording

Facial EMG data were measured by 0.7 cm in diameter Ag/AgCl electrodes (Prokidai, Sagara, Japan) with a 1.5-cm inter-electrode spacing), a PowerLab 16/35 data acquisition system, an EMG-025 amplifier (Harada Electronic Industry, Sapporo, Japan and LabChart Pro v8.0 software (ADInstruments, Dunedin, New Zealand). The data were filtered online with a band-pass of 20–400 Hz and digitized at a sampling rate of 1000 Hz. According to guidelines [23,24] and previous studies [25,26], electrodes were placed on the corrugator supercilii, zygomatic major, masseter, and suprahyoid muscles (a ground electrode was placed on the forehead).

The SCR data were measured by 1.0 cm in diameter Ag/AgCl electrodes (Vitrode F; Nihonkoden, Tokyo, Japan), a Model 2701 BioDerm Skin Conductance Meter (UFI, Morro Bay, CA, USA), and the same data acquisition system and software used for the EMG recording. The data were sampled at 1000 Hz. According to guidelines [27,28], the electrodes were attached to the palmar surface of the medial phalanges of the left index and middle fingers. We also recorded HR via the same apparatus as used for the SCR recording. According to guidelines for electrocardiography [29], electrodes were placed on the left wrist and left ankle. Beats were automatically calculated per minute by the software, which was sampled at 1000 Hz.

Nose-tip temperature data were measured via the FLIR A655sc infrared thermal imaging camera and Research IR Max v4.40 software (FLIR Systems, Wilsonville, OR, USA). The camera was set to capture the entire face of each participant, placing next to the computer screen. The data were sampled at 50 Hz with a spatial resolution of 640 horizontal × 480 vertical pixels.

To monitor motion and the x-, y-, z-axial acceleration measures, an accelerometer (Bio-research Center, Nagoya, Japan) was attached to the jaw. A digital web camera (HD1080P; Logicool, Tokyo, Japan) recorded unobtrusively, but these signals were not analyzed.

### 2.5. Data Analysis

#### 2.5.1. Preprocessing

Psychophysiological Analysis Software 3.3 (Computational Neuroscience Laboratory, the Salk Institute, LA Jolla, CA, USA) and in-house programs on MATLAB 2020a (MathWorks, Natick, MA, USA) analyzed the EMG data, which were sampled during the baseline period for 0.5 s immediately before the period of food consumption and during the period of food consumption for 10 s during each trial. The data for each trial were rectified, baseline-corrected to the average value over the baseline period, and averaged over the period of food consumption. Then, the values for each stimulus were standardized to z scores for each participant. The maximum SCR value during 1.5–10 s of the period of consuming food was calculated for each trial and standardized for each participant. For each trial, the baseline-corrected, averaged, and standardized HR values were calculated, but the data were not rectified. Research IR Max v4.40 software (FLIR Systems) analyzed the thermal images of the nose-tip temperature data. The data were extracted from a 3 × 3-pixel region of interest located on the nose tip, as in a previous study [8]. Then, the data were baseline-corrected, averaged, and standardized as in the case of the analysis of HR.

#### 2.5.2. Statistical Analysis

To estimate subjective-physiological emotional coherence, we analyzed intra-individual correlations between subjective ratings and EMG activity, and compared the correlation coefficients of older and young adults [9]. For each participant, Pearson’s product-moment correlation coefficients (*r*-values) were calculated between the subjective ratings and the physiological signals to examine individual-level coherence between subjective experiences and physiological activity. The *r*-values were normalized using the Fisher transformation, and analyzed first by the one-sample *t*-test (two-tailed), to test for significant mean differences from zero in each group. Then, the group differences were tested using an independent *t*-test (two-tailed). This two-stage random-effects analysis can reveal the generalizability across individuals [30]. The data of one young participant, who frequently changed their head position, preventing analysis of the thermal images, were excluded from analyses. We made a priori predictions and analyzed the relationships between the ratings of liking/wanting/valence and EMG measured from the corrugator supercilii/masseter/suprahyoid muscles. No adjustment for multiple testing [31,32] was conducted, as these analyses were planned and conducted independently. The statistical threshold was set at *p* < 0.05. We also explored testing other relationships in the same manner, with a more conservative threshold of *p* < 0.001. We depicted the group-mean values and regression lines for selected subjective-physiological relationships to illustrate the relationships visually between the subjective ratings and physiological responses at the group level.

## 3. Results

**Figure 1:**
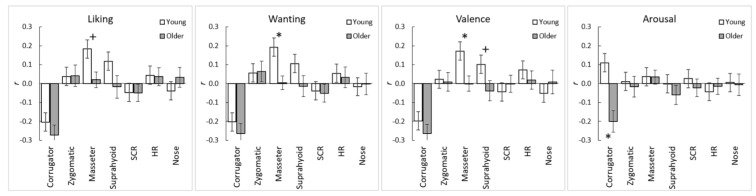
Mean (with *SE*) of intra-individual correlation coefficients between subjective ratings and the physiological measurements across the stimuli in the young and older adult groups. Asterisks indicate significant group differences (*, *p* < 0.05; +, *p* < 0.10). Corrugator = corrugator supercilia; Zygomatic = zygomatic major; SCR = skin conductance response; HR = heart rate; Nose = nose-tip temperature.

Mean ± SD of subjective ratings and physiological activity in response to each stimulus for both age groups are presented in the Supplementary Material (Tables S1–S4). The mean ± Standard error (SE) data of the overall subjective ratings and physiological activity for each hedonic quality category (negative, neutral, positive), with related results, are demonstrated in the (Supplementary Material Figures S1 and S2). For each participant, the correlation coefficients (*r*-values) between the ratings and the physiological signals during food intake were calculated to examine intra-individual subjective-physiological coherence during food intake (Figure 1).

First, the *r*-values after performing the Fisher z transformation were subjected to one-sample *t*-tests against a value of zero for each group (Table 1). In the young adult group, the subjective ratings of liking, wanting, and valence were significantly negatively correlated with corrugator EMG activity (*t* > 3.79, *p* < 0.001, *d* > 0.70) and positively correlated with masseter EMG activity (*t* > 2.41, *p* < 0.05, *d* > 0.44). The suprahyoid EMG was also positively correlated with the liking rating (*t* = 2.20, *p* = 0.037, *d* = 0.41).

Similar to the young adult group, the subjective ratings of liking, wanting, and valence in the older adult group were significantly negatively correlated with corrugator EMG activity (*t* > 4.60, *p* < 0.001, *d* > 0.89). In this group, the arousal ratings were also significantly negatively correlated with corrugator supercilii EMG activity (*t* = 3.45, *p* = 0.002, *d* = 0.66). No other significant associations between subjective ratings and physiological activity were detected in either group (*p* > 0.05).

**Figure 2:**
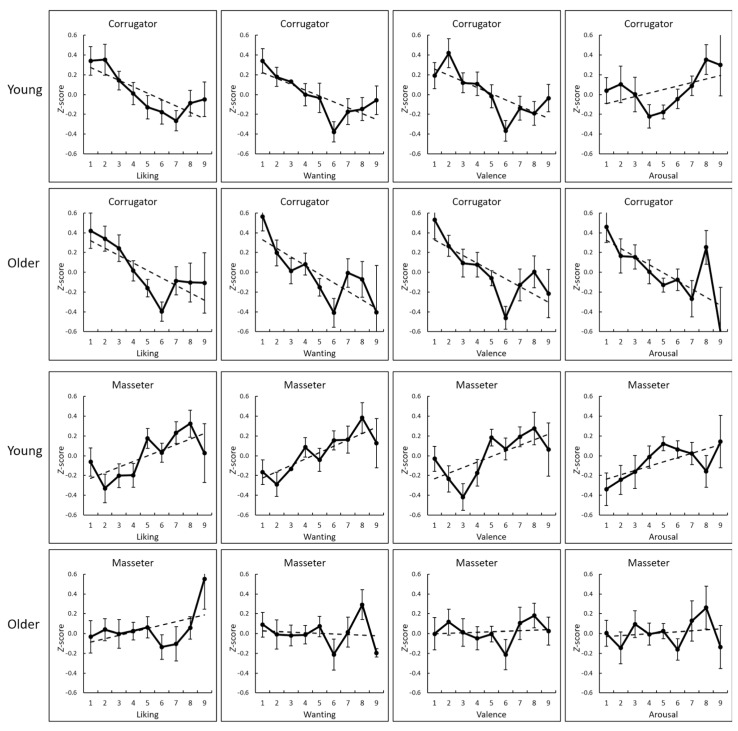
Group mean (with SE) values and regression lines of the subjective ratings (liking, wanting, and valence) and electromyography activity of the corrugator supercilii and masseter muscles in the young and older adult groups (standardized for each individual).

Next, the *z* transformed *r*-values were analyzed for group comparisons (Table 2). The results revealed significant group differences in the wanting-masseter EMG and valence-masseter EMG correlations (*t* > 2.09, *p* < 0.05, *d* > 0.55), with a stronger positive correlation observed in the young than the older adult group. Significant group differences were observed in the direction of coherence for the pair of arousal ratings and corrugator EMG activity (*t* = 3.64, *p* < 0.001, *d* = 0.97). No other significant group differences were observed (*p* > 0.05). Figure 2 illustrates the group-mean relationships between the subjective ratings and the corrugator and masseter EMG activities in each group.

## 4. Discussion

Coherent reactions to emotion-evocative stimuli involving the mind and body have been suggested to promote psychological and physical well-being [1,2,3,4], and young adults have been reported to demonstrate subjective-physiological emotional coherence during food consumption [8,9]. Although related research on older adults has pointed to a critical role of subjective-physiological emotional coherence in good nutrition, no studies have directly examined subjective-physiological emotional coherence during food consumption in older populations. In this study, we compared subjective-physiological emotional coherence during food consumption between older and young adults. The participants consumed bite-sized gel-type foods of different flavors and provided subjective ratings of the foods while their physiological responses were simultaneously measured. The key findings of the study were that (1) intra-individual correlation analyses demonstrated the ratings of liking, wanting, and valence were negatively correlated with corrugator EMG activity both in the older and young groups, indicating no age differences in subjective-physiological emotional coherence between these hedonic ratings and corrugator EMG activity; (2) the positive correlations between the wanting and valence ratings and masseter EMG activity among the older adult group were significantly weaker than those of the young adult group; (3) the arousal ratings were negatively correlated with corrugator EMG activity in the older adult group only.

Consistent with our hypothesis, the liking, wanting, and valence ratings of the foods were negatively correlated with corrugator EMG activity in the older adults, and the degree of subjective-physiological emotional coherence was comparable to that of the young adults, suggesting the maintained subjective-physiological emotional coherence between hedonic experiences and the corrugator EMG activity during food consumption in older populations. Participants demonstrated increased or decreased corrugator EMG activity depending on whether consuming the foods was unpalatable or palatable, regardless of age. These coordinated responses between the subjective hedonic experience and corrugator muscle activity are in accord with the claim that corrugator muscle activity reflects the dimensional aspect of emotions [33] and previous reports of coherence studies of young adults using emotional film clips, which demonstrated that corrugator muscle activity is associated with the hedonic aspect of subjective experience [34,35,36]. Furthermore, the results of the present study are consistent with a previous study that examined the relationship between subjective ratings of odors and corrugator EMG activity in older adults [7]. Olfaction is intimately associated with tasting food because it plays a major role in food pleasure [7]. In their study, similarly to the young adults, older adults demonstrated negative correlations between odor pleasantness and corrugator EMG activity, with higher corrugator EMG activities to aversive smells. Similarly, our results are consistent with relevant age-group comparative research on the coherence between subjective ratings and corrugator EMG, with no age effects reported [10].

Consistent with the prediction in the introduction, we found weaker correlations of wanting and valence ratings with masseter EMG among older adults than their young counterparts. It is conceivable that such differences might have been derived from the decrease in chewing efficiency among older adults, due to a decline in masticatory muscle mass and/or tooth loss associated with increasing age [15,16,17]. This age-relevant physical factor might have prevented older adults from demonstrating an increase/decrease in masseter EMG activity following the pleasantness of the food, leading to weakened subjective-physiological emotional coherence for the associations between wanting and valence ratings and masseter EMG among older adults. Because we did not control for the possible influence of lower chewing efficacy on the magnitude of emotional coherence between subjective ratings and masseter EMG activity in older adults, the exact relationship between subjective experience and masseter EMG activity was not fully elucidated. Additional studies will be needed to clarify the mechanisms by which age differences in the coherence between these hedonic ratings and masseter EMG activity emerged.

An unexpected result was that the arousal ratings were negatively correlated with corrugator EMG activity in the older adult group only; positively tasted foods with heightened arousal appeared to be associated with decreased eyebrow activity only among the older adults. This pattern of results is rare but was indeed demonstrated in a previous study, in which the decreased activity of the corrugator muscle was associated with a moderately heightened level of arousal to positive stimuli [37]. More importantly, our results are consistent with a previous study examining subjective-physiological emotional coherence in older adults using emotion-eliciting films as stimuli, in which the older adults revealed greater emotional coherence assessed by an arousal-based emotional subjective experience [10], though the measured physiological signals differed.

Maintained greater emotional coherence of subjective experiences (based on hedonic and arousal ratings) and corrugator EMG activity among older adults provide theoretical and methodological implications for understanding emotion processing in older populations. Emotions are assumed to unfold in concert with the coordinated responses between subjective and physiological responses to emotional stimuli, with the former being shaped by the latter [2,38,39,40]. These considerations imply that subjective-physiological (mind-body) coherence in emotion is a fundamental constituent of the function of emotions. Although there was an assumption of weakened connections between mind-body among older adults [41], our results do not support this hypothesis, on the grounds that older adults demonstrated coherence between subjective ratings and corrugator EMG activity in a fashion comparable to that of young adults. It is therefore possible to say that the essential function of emotions does not decrease as age advances [42,43].

In terms of methodological implications, it has been suggested that the degree of intensity of elicited emotions should be sufficiently high to detect subjective-physiological emotional coherence [10,34], and emotion-inducing stimuli, such as film clips, have been used to effectively evoke targeted emotions [34,35,36,44,45]. Similarly, tasting food is a powerful stimulus domain for evoking affective reactions [46]. Our study suggests that food was an emotion-eliciting stimulus that induced a sufficient level of emotional reactivity, which led, possibly, to an accurate estimate of subjective-physiological emotional coherence. Tasting foods as an emotion-inducing stimulus, therefore, should be recognized as a novel and effective tool for measuring subjective-physiological emotional coherence, to further understand emotion processing in older adults.

The practical implications of this study are the relationship between subjective-physiological emotional coherence and quality of health and life among older adults. It has been demonstrated that older adults whose hedonic responses to disgusting smells were decoupled with increased corrugator EMG activity had the poorest nutritional status, suggesting that subjective-physiological emotional coherence plays a critical role in the consumption of appropriate foods in older adults [7]. In our study, older adults aged over 65 years without serious cognitive problems (as assessed by the MMSE), maintained coherence between subjective hedonic responses and corrugator EMG activity to foods. Considering the ties between nutritional status and the coupling of hedonic experiences and corrugator activities, our results imply that healthy older adults are capable of maintaining better nutritional status if they maintain coherence between subjective feelings and corrugator activity. There has been an increasing need to optimize nutritional status and maintain good health among older adults as decreased nutritional intake due to a poor appetite is assumed to be associated with increasing age [47]. Maintaining emotional coherence between subjective and physiological reactivity to foods among older adults might help to maintain better nutritional status, thereby contributing to the quality of health and life among older adults. Our results also imply that assessing facial corrugator EMG activity to complement subjective hedonic responses to foods could be beneficial for older patients who have difficulty reporting accurate subjective experiences, given the importance of the positive influence of food on emotions [48].

Finally, we should acknowledge the limitations of our study. First, we did not assess taste/flavor acuity in older adults. Therefore, it remains likely that decreased sensitivity to the taste of foods might have contributed to the degree of subjective-physiological emotional coherence in older adults, given that the reduction of taste acuity has been well documented in older adults [6,11,12,13]. Future research should address this limitation to achieve a comprehensive understanding of the relationship between taste acuity and subjective-physiological emotional coherence during food consumption in later adulthood. Second, although some authors argue the significance of subjective-physiological emotional coherence in terms of psychological [1,2,3] and physical [4] well-being, we did not directly examine the relationship between subjective-physiological emotional coherence and well-being in older adults as they consumed food, which should be investigated in future studies. Third, for simplicity, each food sample contained a single flavor component. Although the flavor compounds used in this study are consumed as part of a normal diet, normal foods often contain mixtures of flavor compounds. Therefore, using normal foods as stimuli will be important in future studies to determine the ecological validity of the present results. Finally, only normal-weight participants were tested in this study because there have been several reports that obese participants exhibit patterns of behavioral and neural emotional processing in response to food that differ from those of normal-weight participants [49,50]. It is therefore possible that the relationship between subjective-physiological emotional coherence during food consumption and age varies according to body weight. Studies including participants with a high BMI are warranted to clarify the psychophysiological mechanisms underlying obesity.

## 5. Conclusions

While the importance of subjective-physiological emotional coherence for psychological well-being and nutrition status has been suggested in previous research, there has been no studies examining subjective-physiological emotional coherence among older adults while they consume food. The present study revealed that emotional coherence between subjective experiences and corrugator EMG activity during food consumption was maintained or increased in older compared to young adults. These results suggest that the essential function of emotions is maintained in older adults, which probably enhances the quality of health and life in late adulthood. Further study will be needed for a deeper understanding of the relationship between subjective-physiological emotional coherence and food intake during late adulthood.

## Figures and Tables

**Table 1:** Results of one-sample *t*-tests (vs. 0; two-tailed) for subjective-physiological coherence in young and older groups.

Group	Subjective	Statistic	Physiological						
			Corrugator	Zygomatic	Masseter	Suprahyoid	SCR	HR	Nose
Young	Liking	*t*	**4.11**	0.51	**2.48**	**2.20**	1.00	0.97	1.00
		*p*	**<0.001**	0.613	**0.019**	**0.037**	0.328	0.340	0.325
		*d*	**0.76**	0.10	**0.46**	**0.41**	0.19	0.18	0.19
	Wanting	*t*	**4.24**	0.85	**2.78**	2.04	0.78	1.17	0.96
		*p*	**<0.001**	0.403	**0.010**	0.051	0.441	0.253	0.345
		*d*	**0.79**	0.16	**0.52**	0.38	0.15	0.22	0.18
	Valence	*t*	**3.79**	0.36	**2.41**	1.89	0.91	1.51	0.33
		*p*	**<0.001**	0.724	**0.023**	0.07	0.37	0.143	0.742
		*d*	**0.70**	0.07	**0.45**	0.35	0.17	0.28	0.06
	Arousal	*t*	1.81	0.20	0.54	0.03	0.60	1.20	0.93
		*p*	0.081	0.843	0.592	0.977	0.556	0.24	0.359
		*d*	0.34	0.04	0.10	0.01	0.11	0.22	0.17
Older	Liking	*t*	**4.98**	0.71	0.43	0.15	1.07	0.80	0.68
		*p*	**<0.001**	0.486	0.671	0.882	0.294	0.432	0.504
		*d*	**0.96**	0.14	0.08	0.03	0.21	0.15	0.13
	Wanting	*t*	**4.60**	1.16	0.08	0.13	1.02	0.64	0.01
		*p*	**<0.001**	0.256	0.94	0.897	0.316	0.531	0.989
		*d*	**0.89**	0.22	0.02	0.03	0.20	0.12	0.00
	Valence	*t*	**4.94**	0.16	0.06	0.61	0.01	0.42	0.25
		*p*	**<0.001**	0.875	0.949	0.547	0.989	0.679	0.805
		*d*	**0.95**	0.03	0.01	0.12	0.00	0.08	0.05
	Arousal	*t*	**3.45**	0.32	0.90	1.08	0.51	0.35	0.08
		*p*	**0.002**	0.754	0.374	0.291	0.615	0.733	0.94
		*d*	**0.66**	0.06	0.17	0.21	0.10	0.07	0.02

Degrees of freedom were 28 and 26 for the young and older adult groups, respectively, except for the young nose data (i.e., 27). Significant results (*p* < 0.05) are in bold. Corrugator = corrugator supercilii; Zygomatic = zygomatic major; SCR = skin conductance response; HR = heart rate; Nose = nose-tip temperature.

**Table 2:** Results of the independent *t*-tests (young vs. older adults; two-tailed) for subjective-physiological coherence.

Subjective	Statistic	Physiological						
		Corrugator	Zygomatic	Masseter	Suprahyoid	SCR	HR	Nose
Liking	*t*	0.97	0.06	1.92	1.58	0.05	0.11	1.02
	*p*	0.339	0.95	0.061	0.12	0.959	0.915	0.313
	*d*	0.26	0.02	0.51	0.42	0.01	0.03	0.27
Wanting	*t*	0.90	0.08	**2.40**	1.51	0.12	0.27	0.92
	*p*	0.373	0.937	**0.02**	0.138	0.904	0.791	0.361
	*d*	0.24	0.02	**0.64**	0.40	0.03	0.07	0.25
Valence	*t*	0.90	0.19	**2.09**	1.77	0.66	0.77	0.40
	*p*	0.374	0.85	**0.041**	0.082	0.51	0.446	0.694
	*d*	0.24	0.05	**0.56**	0.47	0.18	0.21	0.11
Arousal	*t*	**3.64**	0.37	0.02	0.82	0.78	0.54	0.90
	*p*	**<0.001**	0.712	0.985	0.416	0.439	0.595	0.371
	*d*	**0.97**	0.10	0.01	0.22	0.21	0.14	0.24

Degrees of freedom were 54 for all except the nose data.(i.e., 53). Significant results (*p* < 0.05) are in bold. Corrugator = corrugator supercilii; Zygomatic = zygomatic major; SCR = skin conductance response; HR = heart rate; Nose = nose-tip temperature.

## Data Availability

Datasets are available from the corresponding author upon request.

## Supplementary Materials

The following supporting information can be downloaded at: https://www.mdpi.com/article/10.3390/nu14224736/s1, Figure S1: Mean ± SE data of the overall subjective ratings for each emotion category in each age group; Figure S2: Mean ± SE data of the overall physiological activity for each emotion category in each age group. Supplementary Results: analysis of subjective and physiological responses; Table S1: Mean (with SD) subjective ratings for each stimulus category in the older group; Table S2: Mean (with SD) physiological activity for each stimulus category in the older group; Table S3: Mean (with SD) subjective ratings for each stimulus category in the young adult group; Table S4: Mean (with SD) physiological activity for each stimulus category in the young adult group.
