# Supplementary material for: Subjective-Physiological Coherence during Food Consumption in Older Adults
Source: Nutrients. 2022 Nov 9;14(22):4736. doi: 10.3390/nu14224736 (PMC9698163; doi:10.3390/nu14224736)
Supplement: Supplementary file 1 [file nutrients-14-04736-s001.zip › nutrients-1992355-supplementary.pdf]

nutrients
MDPI

Supplementary Table S1. Mean (with Standard Deviation (SD)) subjective ratings for each stimulus category in the older group.

Stimulus	Measure			
	Liking	Wanting	Valence	Arousal
Vanillin	5.3	5.1	5.4	5.3
	(1.8)	(1.8)	(1.7)	(1.4)
Maltol	5.9	5.8	5.7	5.7
	(1.3)	(1.4)	(1.7)	(1.3)
Ethyl butyrate	5.9	5.7	5.5	5.3
	(1.4)	(1.5)	(1.9)	(1.4)
Phenethyl alcohol	4.6	4.5	4.3	4.7
	(1.5)	(1.5)	(1.6)	(1.4)
Acetoin	4.1	4.0	4.3	4.3
	(1.6)	(1.6)	(1.6)	(1.4)
2,5−Dimethylpyrazine	4.0	3.7	3.9	4.1
	(1.4)	(1.3)	(1.4)	(1.6)
Isovaleric acid	4.0	3.7	3.7	4.2
	(1.8)	(1.6)	(1.7)	(1.7)
2−Nonenal	3.0	2.8	3.0	3.5
	(1.6)	(1.5)	(1.4)	(1.8)
Indole	4.2	4.1	4.2	4.4
	(1.7)	(1.6)	(1.7)	(1.6)

Supplementary Table S2. Mean (with SD) physiological activity for each stimulus category in the older group.

Stimulus	Measure						
	Corrugator	Zygomatic	Masseter	Suprahyoid	SCR	HR	Nose
Vanillin	−0.4	0.0	0.2	0.0	0.1	0.2	0.0
	(0.8)	(0.8)	(0.6)	(0.6)	(0.7)	(0.7)	(0.8)
Maltol	−0.1	0.0	−0.1	0.1	0.0	0.1	0.0
	(0.6)	(0.6)	(0.6)	(0.7)	(0.6)	(0.7)	(0.6)
Ethyl butyrate	−0.3	0.1	0.0	−0.1	−0.1	−0.2	0.0
	(0.6)	(0.8)	(0.9)	(0.8)	(0.6)	(0.7)	(0.8)
Phenethyl alcohol	−0.1	−0.1	0.0	−0.2	0.0	0.0	−0.1
	(0.6)	(0.7)	(0.4)	(0.6)	(0.7)	(0.7)	(0.8)
Acetoin	−0.1	−0.2	−0.1	0.1	−0.1	0.0	0.4
	(0.6)	(0.8)	(0.6)	(0.6)	(0.5)	(0.6)	(0.6)
2,5−Dimethylpyrazine	0.1	0.1	0.0	0.1	−0.1	0.0	−0.1
	(0.6)	(0.6)	(0.7)	(0.6)	(0.6)	(0.6)	(0.8)
Isovaleric acid	0.3	0.3	0.0	0.1	0.0	0.0	0.2
	(0.6)	(0.6)	(0.6)	(0.6)	(0.6)	(0.5)	(0.7)
2−Nonenal	0.4	0.0	0.2	0.0	0.0	−0.1	−0.1
	(0.8)	(0.6)	(0.5)	(0.8)	(0.7)	(0.8)	(0.6)
Indole	0.0	−0.2	−0.2	−0.1	0.1	0.0	−0.3
	(0.5)	(0.8)	(0.6)	(0.8)	(0.6)	(0.7)	(0.9)

Supplementary Table S3. Mean (with SD) subjective ratings for each stimulus category in the young adult group.

Stimulus	Measure			
	Liking	Wanting	Valence	Arousal
Vanillin	6.5	6.1	6.6	5.8
	(1.3)	(1.7)	(1.2)	(0.9)
Maltol	6.4	6.2	6.4	5.5
	(1.2)	(1.2)	(1.0)	(1.3)
Ethyl butyrate	6.5	6.2	6.5	5.9
	(1.4)	(1.9)	(1.4)	(1.1)
Phenethyl alcohol	4.3	3.8	4.2	5.0
	(2.0)	(1.9)	(2.0)	(1.9)
Acetoin	4.8	4.5	4.8	5.0
	(1.8)	(1.7)	(1.7)	(1.5)
2,5−Dimethylpyrazine	3.7	3.2	3.5	5.0
	(1.5)	(1.5)	(1.5)	(1.9)
Isovaleric acid	3.9	3.4	3.6	4.9
	(1.6)	(1.7)	(1.5)	(1.8)
2−Nonenal	2.8	2.4	2.6	5.5
	(1.7)	(1.5)	(1.6)	(2.1)
Indole	3.7	3.2	3.8	4.9
	(1.7)	(1.7)	(1.7)	(2.0)

Supplementary Table S4. Mean (with SD) physiological activity for each stimulus category in the young adult group.

Stimulus	Measure						
	Corrugator	Zygomatic	Masseter	Suprahyoid	SCR	HR	Nose
Vanillin	−0.3	0.0	0.0	0.1	−0.2	0.0	0.0
	(0.5)	(0.4)	(0.6)	(0.7)	(0.6)	(0.7)	(0.6)
Maltol	−0.1	0.1	0.1	0.1	0.0	0.1	0.4
	(0.7)	(0.5)	(0.5)	(0.6)	(0.8)	(0.6)	(0.7)
Ethyl butyrate	0.0	0.2	0.4	0.3	0.0	−0.2	−0.2
	(0.6)	(0.8)	(0.8)	(0.5)	(0.6)	(0.8)	(0.6)
Phenethyl alcohol	0.0	0.0	−0.1	0.0	0.0	0.1	−0.2
	(0.6)	(0.6)	(0.6)	(0.5)	(0.5)	(0.7)	(0.8)
Acetoin	0.0	0.0	0.0	0.0	0.0	0.1	0.2
	(0.6)	(0.5)	(0.6)	(0.6)	(0.7)	(0.7)	(0.7)
2,5−Dimethylpyrazine	0.2	0.0	0.1	−0.1	0.0	0.1	−0.2
	(0.6)	(0.7)	(0.7)	(0.7)	(0.6)	(0.6)	(0.8)
Isovaleric acid	−0.1	−0.1	−0.2	−0.1	−0.1	−0.2	0.2
	(0.7)	(0.7)	(0.6)	(0.8)	(0.7)	(0.8)	(0.5)
2−Nonenal	0.2	−0.1	−0.2	−0.1	0.3	0.1	0.1
	(0.7)	(0.8)	(0.8)	(0.7)	(0.8)	(0.7)	(0.8)
Indole	0.1	0.0	−0.1	−0.1	0.0	−0.1	0.0
	(0.8)	(0.7)	(0.7)	(0.9)	(0.6)	(0.6)	(0.8)

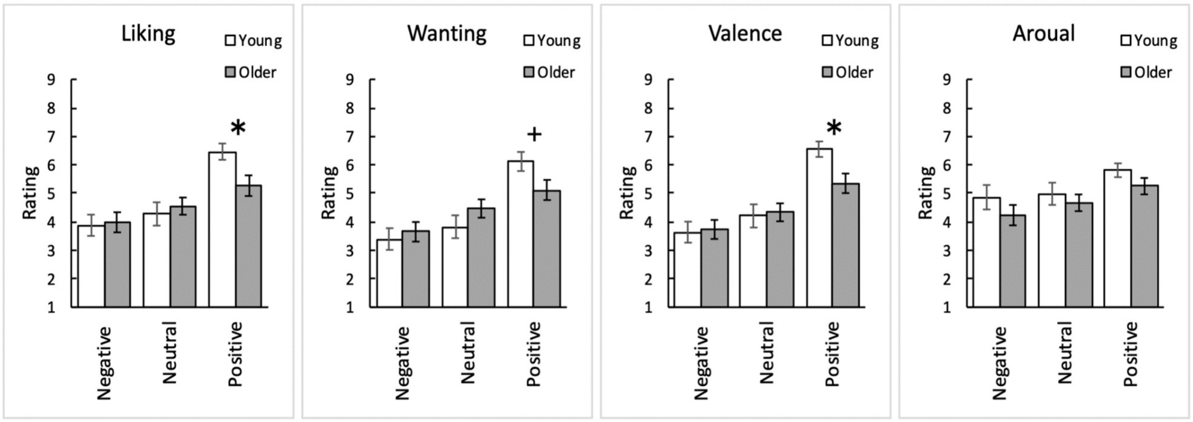

Supplementary Figure S1. Mean ± SE data of the overall subjective ratings for each emotion category in each age group. Asterisks indicate significant group differences (*, $p < 0.05$; +, $p < 0.10$).

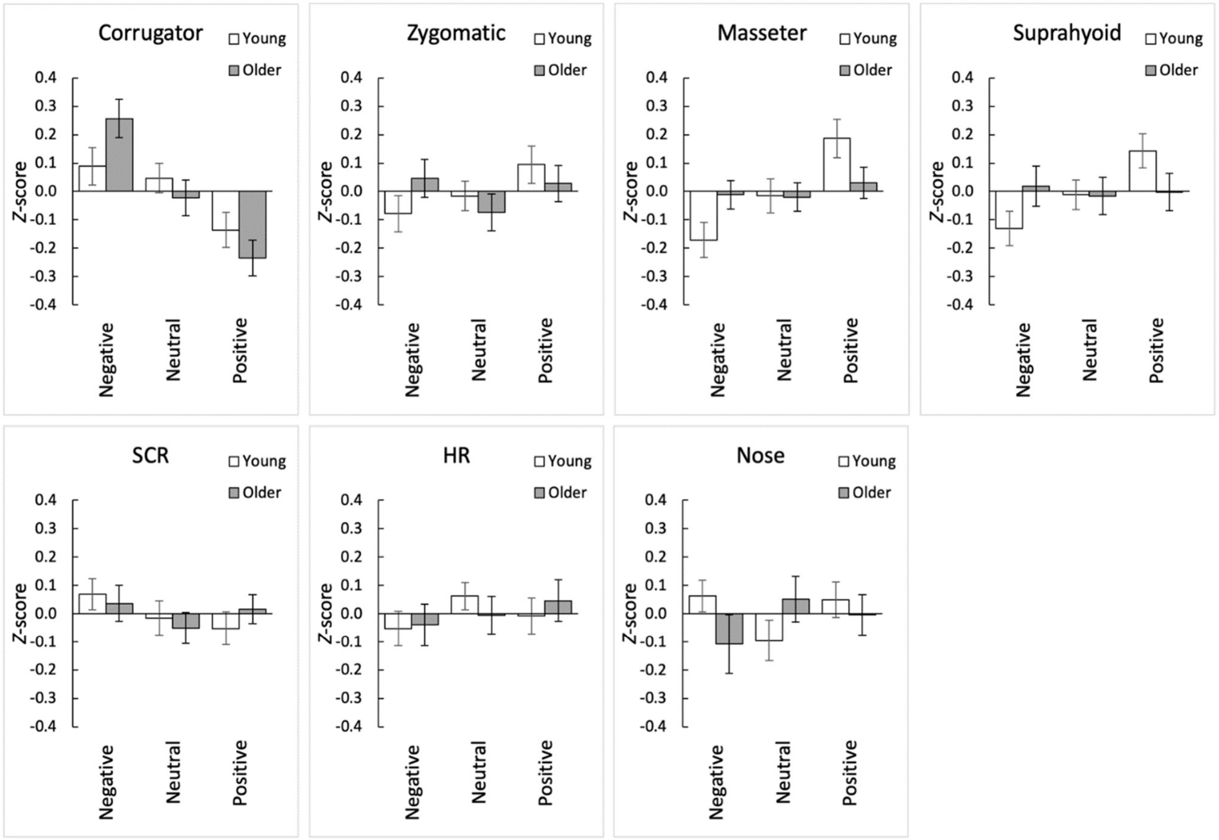

Supplementary Figure S2. Mean ± SE data of the overall physiological activity for each emotion category in each age group. Supplementary Results: analysis of subjective and physiological responses.

To investigate the subjective and physiological responses separately, two−way analysis of variance (ANOVA) was performed with the age group (older and young adults) as the between−participant factor and hedonic quality of the

food stimuli (negative, neutral, positive) as the within–participant factor. Follow–up analyses of significant interactions for the simple effects were conducted.

Liking ratings

Two–way ANOVA with age group and hedonic quality revealed a significant interaction between age group and hedonic quality ($F(2, 108) = 4.49$, $p = 0.013$, $\eta^2 p = 0.077$), as well as a significant main effect of hedonic quality ($F(2, 108) = 128.53$, $p < 0.001$, $\eta^2 p = 0.704$). No significant main effect of age group was observed ($F(1,54) = 1.268$, $p = 0.265$). Follow–up analyses of the significant interaction revealed that the simple main effect of age on hedonic quality was significant for positive stimuli ($F(1, 162) = 6.83$, $p = 0.010$), where young adults had higher liking ratings of positive food stimuli, compared to older adults, indicating that the young adults preferred the food stimuli that elicited positive feelings, as compared to the older adults.

Wanting ratings

Two–way ANOVA revealed a significant interaction between age group and hedonic quality ($F(2, 108) = 4.75$, = 0.011, $\eta^2 p = 0.081$), as well as a significant main effect of hedonic quality ($F(2, 108) = 125.86$, $p < 0.001$, $\eta^2 p = 0.700$). The main effect of age group was not significant ($F(1,54) = 0.029$, $p = 0.864$). The simple main effect of age on hedonic quality was marginally significant for positive stimuli ($F(1, 162) = 4.00$, $p = 0.050$). These results indicate that the young adults tended to want to eat more of the food stimuli that elicited positive feelings compared to the older adults.

Valence ratings

Two–way ANOVA with age group and hedonic quality revealed a significant interaction between age group and hedonic quality ($F(2, 108) = 6.96$, $p = 0.001$, $\eta^2 p = 0.114$), as well as a significant main effect of hedonic quality ($F(2, 108) = 122.18$, $p < 0.001$, $\eta^2 p = 0.693$). No significant main effect of age group was observed ($F(1,54) = 0.227$, $p = 0.634$). Follow–up analyses of the significant interaction revealed that the simple main effect of age on hedonic quality was significant for positive stimuli ($F(1, 162) = 7.03$, $p = 0.001$), where the young adults had higher valance ratings for positive food stimuli, as compared to the older adults, indicating that the young adults rated the food stimuli that elicited positive feelings more favorably than did the older adults.

Arousal ratings

Two–way ANOVA with age group and hedonic quality revealed significant main effects of hedonic quality ($F(2, 108) = 19.08$, $p < 0.001$, $\eta^2 p = 0.261$) and age group ($F(1,54) = 5.043$, $p = 0.029$, $\eta^2 p = 0.085$). No significant interaction between age group and hedonic quality was detected ($F(2, 108) = 2.18$, $p = 0.118$).

Corrugator electromyography (EMG)

Two–way ANOVA revealed a significant main effect of hedonic quality ($F(2, 108) = 10.45$, $p < 0.001$, $\eta^2 p = 0.162$). No significant interaction between age group and hedonic quality ($F(2, 108) = 1.58$, $p = 0.210$) was detected, and no significant main effect of age group was observed ($F(1, 54) = 0.00$, $p = 0.973$).

Zygomatic EMG

No significant interaction between age group and hedonic quality was observed ($F(2, 108) = 0.89$, $p = 0.412$), and no significant main effects of hedonic quality ($F(2, 108) = 1.03$, $p = 0.360$), or age group were detected ($F(1, 54) = 0.00$, $p = 1.000$).

Masseter EMG

Two−way ANOVA revealed a significant main effect of hedonic quality ($F(2, 108) = 3.70$, $p = 0.028$). No significant interaction between age group and hedonic quality was observed ($F(2, 108) = 2.57$, $p = 0.081$), and no significant main effect of age group was detected ($F(1, 54) = 0.00$, $p = 1.000$).

Suprahyoid EMG

No significant interaction was observed between age group and hedonic quality ($F(2, 108) = 2.02$, $p = 0.138$), and no significant main effects of hedonic quality ($F(2, 108) = 1.35$, $p = 0.263$), or age group were detected ($F(1, 54) = 2.33$, $p = 0.133$).

SCR

No significant interaction was observed between age group and hedonic quality ($F(2, 108) = 0.23$, $p = 0.795$), and no significant main effects of hedonic quality ($F(2, 108) = 0.98$, $p = 0.377$), or age group were observed ($F(1, 54) = 0.00$, $p = 0.963$).

HR

No significant interaction was observed between age group and hedonic quality ($F(2, 108) = 0.29$, $p = 0.753$), and no significant main effects of hedonic quality ($F(2, 108) = 0.44$, $p = 0.643$), or age group were found ($F(1, 54) = 0.00$, $p = 0.989$).

Nose temperature

No significant interaction was observed between age group and hedonic quality ($F(2, 108) = 1.06$, $p = 0.352$), and no significant main effects of hedonic quality ($F(2, 108) = 0.18$, $p = 0.838$), or age group were detected ($F(1, 54) = 1.01$, $p = 0.320$).
